# School-based nutrition interventions for Indigenous children in Canada: a scoping review

**DOI:** 10.1186/s12889-019-8120-3

**Published:** 2020-01-06

**Authors:** Christina Gillies, Rosanne Blanchet, Rebecca Gokiert, Anna Farmer, Jessica Thorlakson, Laura Hamonic, Noreen D. Willows

**Affiliations:** 1 Department of Agricultural, Food & Nutritional Science, University of Alberta, 11405 87 Avenue, Edmonton, AB T6G 2P5 Canada; 2 Faculty of Extension, University of Alberta, 10230 Jasper Avenue, Edmonton, AB T5J 4P6 Canada; 3 University of Alberta Library, University of Alberta, Edmonton, AB Canada

**Keywords:** Indigenous, First nations, Inuit, Metis, School, Nutrition, Scoping review, Intervention

## Abstract

**Background:**

Indigenous children in Canada (First Nations, Inuit, and Métis) are disproportionately affected by nutrition-related chronic diseases such as obesity and diabetes. Comprehensive school-based nutrition interventions offer a promising strategy for improving children’s access to healthy foods and sustaining positive eating behaviors. However, little is known about school-based nutrition interventions for Indigenous children. The objectives of this scoping review were to identify school-based nutrition interventions for Indigenous children in Canada and describe their components.

**Methods:**

The scoping review consisted of searches in seven peer-reviewed databases and a general web search for grey literature. Eligibility criteria were applied by two reviewers, and data were extracted and charted by one reviewer using components of the comprehensive school health approach (social and physical environment, teaching and learning, policy, partnerships and services) and additional components with relevance to Indigenous interventions (cultural content, Indigenous control and ownership, funding source, evaluation). Numerical and descriptive summaries were used to present findings.

**Results:**

Thirty-four unique interventions met the inclusion criteria. The majority (97%) of interventions targeted the social and physical environment, most often by offering food programs. Over half of interventions also incorporated teaching and learning (56%) and partnerships and services (59%), but fewer included a policy component (38%). Many interventions included a cultural component (56%) and most (62%) were owned and controlled by Indigenous communities (62%). Finally, over half of interventions disclosed their source(s) of funding (59%), but less than half (41%) included an evaluation component.

**Conclusions:**

The review suggests that school-based interventions for Indigenous children can be more comprehensive by incorporating culturally relevant nutrition education and professional development opportunities for teachers, written school nutrition policies, and activities that actively engage families and community members. The continued focus on Indigenous control and ownership and incorporation of content specific to individual communities may enhance cultural relevancy and sustainability of interventions. Furthermore, there is a need to increase intervention evaluation and the sharing of resources related to funding. These recommendations may be used by communities, as well as by researchers and professionals working with communities, in developing comprehensive school-based nutrition interventions to improve the eating behaviors of Indigenous children.

## Background

Indigenous communities in Canada face significant obstacles to healthy eating due to numerous sociocultural and environmental barriers, including a high prevalence of household and community food insecurity [1–3]. High cost and lack of variety and availability of nutrient-dense foods in both urban and geographically remote areas underlie issues in obtaining nutritionally adequate, acceptable, and safe foods for Indigenous children [1, 2]. The term Indigenous collectively refers to the original inhabitants of Canada and their descendants as defined in Section 35 [2] of the *Canadian Constitution Act, 1982* [4]*.* Indigenous peoples include three district groups: First Nations, Inuit, and Métis. Inuit are the original inhabitants of the Arctic regions of Canada. Most continue to live in these regions, including Nunavut, Nunavik, and Nunatsiavut [5]. Métis are a group of Indigenous peoples whose ancestry can be traced back to the intermarriage of European men and First Nations women. The majority of Métis live in metropolitan areas across Canada, with the largest population residing in Manitoba [5]. First Nations peoples are the descendants of the original inhabitants of Canada who do not recognize themselves as ethnically Inuit or Métis. The First Nations population is concentrated in the western provinces of British Columbia, Alberta, Manitoba, and Saskatchewan [5]. First Nations comprise the largest population of Indigenous peoples (977,230 people), followed by Métis (587,545 people), and Inuit (65,025 people) [5].

Indigenous peoples of Canada are linguistically, culturally, and geographically diverse; however, they share similar barriers to healthy eating as their experiences are situated within a larger macro-context of historical colonization, assimilation policies, and forced removal from traditional lands that are unique to Indigenous peoples [6]. For instance, the dispossession and industrialization of traditional lands has resulted in the loss of knowledge and skills related to land-based food practices (e.g., hunting, gathering, and horticulture) and forced dependence on highly processed, nutrient poor market foods [7]. Due in large part to these barriers, the diets of many Indigenous children are energy-dense and low in nutrient-dense foods like fruits and vegetables [8–10]. These poor dietary patterns contribute to a high risk of nutrition-related chronic diseases like obesity, diabetes, and cardiovascular disease [11]. Effective strategies to improve food environments and eating behaviors of Indigenous children that consider the multiple barriers that Indigenous communities face are needed to ensure that Indigenous children are able to attain optimal nutrition and health.

Schools are an important setting to target nutrition interventions to promote and support healthy eating, considering the time that children spend in schools during their formative years [12]. Research supports the positive impact that school-based nutrition interventions – such as breakfast or lunch programs – can have on the diet, learning, and health outcomes of Indigenous children [13–16]. Evidence suggests that comprehensive, multi-component school-based interventions hold greater potential in promoting and supporting positive health changes in the long-term than single-component nutrition interventions [14, 17–22]. Comprehensive School Health (CSH) is an internationally recognized school-based health promotion approach that integrates multiple aspects of the school environment through four mutually reinforcing components (social and physical environments, teaching and learning, school policy, and partnerships and services) into a single intervention. Evidence from evaluations in non-Indigenous populations have demonstrated that CSH interventions have resulted in increased physical activity, improved dietary habits, and decreased rates of obesity and chronic disease among children [23–26]. Comprehensive school-based nutrition interventions may be effective in Indigenous settings by increasing children’s access to healthy foods and sustaining positive eating behaviors [14, 21]. However, there is limited evidence concerning school-based nutrition interventions in Indigenous communities in Canada.

In 2008, ﻿The Assembly of First Nations performed an environmental scan of school nutrition programs and policies for children in First Nation community schools across Canada. Of the 47.9% of schools that responded to the survey (*n* = 303), 86.7% had a school nutrition program (e.g., breakfast, snack, and/or lunch program) and nearly two-thirds (62.3%) had a school nutrition policy [14]. More recent reviews have identified school-based interventions that aim to improve nutrition knowledge, food preferences, and/or health in Indigenous communities; however, these reviews have largely focused on evaluating the effectiveness and impacts of interventions rather than describing their components [21, 27, 28]. Describing the content and scope of interventions is an important next step in developing evidence-based comprehensive school-based nutrition interventions to improve eating behaviors in Indigenous communities. For this reason, a scoping review was conducted to search and consolidate the extent and nature of interventions as well as identify any existing knowledge gaps.

The primary objective of this scoping review was to identify school-based nutrition interventions for Indigenous children in Canada. The second objective was to describe the main components of the identified school-based nutrition interventions for Indigenous children. Overall, this review was intended to identify gaps and provide recommendations for the development of comprehensive school-based nutrition interventions to optimize nutrition and health outcomes for Indigenous children.

## Methods

This scoping review was conducted following the methodological framework developed by Arksey and O’Malley [29] which included identifying relevant sources of evidence, selecting sources of evidence, charting the data, and summarizing the results. The review is reported following the ﻿Preferred Reporting Items for Systematic reviews and Meta-Analyses extension for Scoping Reviews (PRISMA-ScR) (Additional file 4) [30]. A protocol for the review does not exist, and ethics approval was not required as the review relied solely on publicly available information.

### Identifying relevant sources of evidence

With the assistance of a research librarian (JT) at the University of Alberta, an initial search was conducted to develop and refine the search strategy for the scientific literature. Searches were then conducted by two research librarians (JT and LH) using the following databases: Medline (Ovid), ERIC (Ovid), CINAHL Plus with Full Text (EBSCO), Agricultural & Environmental Sciences (Proquest), Academic Search Complete (EBSCO), Bibliography of Native North Americans (EBSCO), and Dissertations and Theses Global (Proquest). Searches employed both controlled vocabularies, such as Medical Subject Headings (MeSH), and keywords representing concepts such as: (Indigenous or Amerindian) AND (schools or kindergarten) AND (nutrition or diet) AND (Alberta or British Columbia). The search for the scientific literature covered articles published between January 1, 2000 and February 25, 2019. No limiters or facets were used, and search strategies were adapted for each database. The MEDLINE Search Strategy is available in Additional file 1.

In an effort to minimize the risk of omitting relevant sources of evidence, one researcher (CG) conducted a search of the grey literature on the Internet using different combinations of key search terms [31]. Grey literature are documents not formally published in academic sources (e.g., peer-reviewed journals), and include information sources such as newspapers, websites, conference proceedings, and unpublished research (e.g., theses) [31]. First, a filter was applied to limit the Google search to the region of Canada and to the English language. Next, the first ten pages of each search’s hits (representing 100 results) were reviewed, using the title and 2–3 lines of text underneath. This number of pages allowed the search to retrieve the most relevant hits while still being a feasible amount to review [31]. Potentially relevant records were ‘bookmarked’ in the web browser and later entered into an Excel spreadsheet for further screening. For each search strategy, the search terms, number of results retrieved and screened, and date of the search (January 30, 2019) were recorded (Additional file 2). The reference lists of all included sources of evidence were hand-searched by one reviewer (CG) to identify additional relevant sources.

### Selecting sources of evidence

Basic eligibility criteria were defined a priori (Table 1) and were based on sources of evidence having a publication status that the reviewers considered recent enough to be relevant, being published in a language that both reviewers could read and containing information that specifically met the research objectives. To test reviewer agreement of eligibility criteria, two reviewers (CG and RB) independently reviewed a random selection of sources of evidence from the scientific (*n* = 10) and grey (n = 10) literature. Their level of agreement was 100%. At this stage, the reviewers determined that the date criteria would not apply to websites. The reviewers felt it was unlikely that a website would be running if it was outdated by over a decade, as many website hosting platforms require a fee for maintenance. Therefore, if a website was available at the time of the review, it was considered eligible for review and selection whether it had a date listed on its pages or not.

**Table 1:** Eligibility criteria

Inclusion criteria	Exclusion criteria
Published after January 1, 2000 (except for websites)	Published before January 1, 2000 (except for websites)
Available in English	Unavailable in English
Targets one or more Indigenous populations in Canada	Does not target one or more Indigenous population in Canada
Evaluates or describes a school-based nutrition intervention that has been implemented	Does not evaluate or describe a school-based nutrition intervention that has been implemented

In the first stage of selection, two reviewers (CG and RB) applied the eligibility criteria to determine the relevancy of sources of evidence identified in the scientific literature. First, the reviewers screened the title and abstracts for relevancy and copies of the full text were obtained for those that appeared to fit the eligibility criteria. If the relevance of a source of evidence was unclear from the abstract, or if reviewers had discrepant assessment at this stage, the full text was obtained. In the second step, each reviewer read the full text of each article to decide whether it should be chosen for inclusion in the review. Discrepancies between reviewers were discussed, and a third opinion (NDW) was sought for two of the scientific literature sources of evidence.

The grey literature search followed a one-step process whereby sources of information were screened by each reviewer in full to determine both relevancy and inclusion. This one-step determination was followed out of necessity, as the majority of sources of evidence were websites that did not have an abstract or table of contents to screen. There was 100% agreement between the reviewers for all grey literature sources of evidence.

### Charting the data

A standardized data charting form (Additional file 3) was developed by one reviewer (CG) and reviewed by the study team for relevance and appropriateness. The form’s extraction fields captured relevant information on intervention characteristics, including: intervention type (single school or multiple school), intervention name, author and year, location, school name, grade(s) served, and target cultural group (i.e., First Nations, Inuit, Métis). The extraction fields also included eight school-based nutrition interventions components: four components of CSH [25, 32] and four additional key components that may be important to interventions in Indigenous communities [21, 27, 28, 33] as described in Table 2.

**Table 2:** School-based nutrition intervention components

**Comprehensive school health (CSH) intervention components**		
Component	Description	Examples
Social and physical environment	The quality of the relationships between students and staff, as well as with families and the wider community. The facilities, amenities, and equipment in and surrounding the school, and the presence of safe, accessible, and supportive healthy food choices for students and community members.	Peer-support and mentoring programs, student cooking classes and community feasts, staff and peers modelling healthy behaviors, healthy eating messages in newsletters and other forms of communication. Food programs that increase access to healthy foods, vending machines and canteens stocked with healthy options, school or community gardens, nutrition awareness campaigns or contests, healthy foods offered at celebrations and fundraisers, visual displays of healthy messages.
Teaching and learning	Formal and informal curriculum and resources, instilling knowledge and skills for students to improve their eating behaviors and health outcomes, and professional development opportunities for staff related to nutrition.	Incorporating healthy diet and nutrition knowledge into classes and curriculum, gardening programs, offering professional development opportunities for teachers.
Policy	Policies, guidelines, and practices that promote and support student nutrition.	Written nutrition policy, offering foods that are consistent with local, provincial, or national guidelines, and prohibiting certain foods from the school environment.
Partnerships and services	The connections between a school and students’ families, and supportive relationships among schools and other community organizations and sectors to advance student nutrition.	Working with local food services and businesses, partnerships among education and health sectors, and the use of community facilities.
**Additional components for Indigenous school-based nutrition interventions**		
Component	Description	Example
Cultural content	Elements that recognize the diversity of Indigenous communities and are relevant to local cultures and contexts.	Incorporation of traditional foods, practices, and ways of learning.
Indigenous control and ownership	Community-driven and community-led elements that promote self-determination.	Community driven programs and equitable collaboration with researchers and other non-community members.
Funding source	The source providing funding to develop, implement, and/or sustain an intervention.	Donations, grants, and research funds.
Evaluation	The collection and analysis of an intervention.	Formative evaluation, process evaluation, and outcome evaluation.

The charting form was pilot tested by two reviewers (CG and RB) with a random sample of 5 sources of evidence from the scientific or grey literature to ensure all relevant data were captured. Eligible sources of evidence were charted independently by one reviewer (CG) using Microsoft Excel. Only data that were relevant to nutrition were charted (i.e., information about physical activity interventions was not charted), consistent with the a priori objectives of the review. Data extracted about the same intervention described in multiple articles were combined in the charting form. As the goal of this review was to provide an overview of the existing literature regardless of quality, a formal appraisal of the methodological quality of sources of evidence included in the review was not performed [29, 30, 34].

### Summarizing the results

Having charted characteristics of the interventions, a numerical and descriptive summary of the charting results was used to present findings. The comprehensiveness and scope of the interventions was described by drawing upon the four components of CSH and the four additional key components of Indigenous school-based nutrition interventions that were included as extraction fields and defined in Table 2.

## Results

**Fig. 1:**
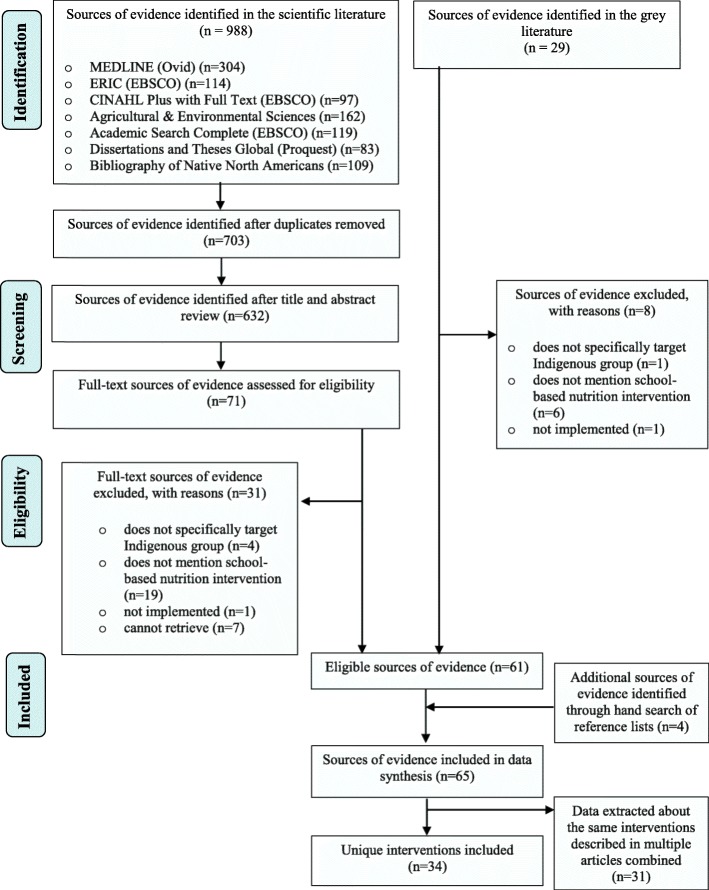
PRISMA flow diagram

A total of 65 sources of evidence were included in the review, representing 34 unique nutrition interventions [13–16, 21, 22, 35–93] (Fig. 1). Of these, 14 (41%) were implemented in a single school and 20 (59%) were implemented in more than one school. Nine interventions (26%) included each of the four components of CSH, and five interventions (15%) included the four additional key components identified as important in school-based nutrition interventions for Indigenous children. Four interventions (12%) included all eight components. Twenty-four interventions targeted First Nations populations (70%), four targeted Inuit populations (12%), and one targeted Métis populations (3%). One intervention (3%) targeted both First Nations and Métis populations. Four interventions (12%) did not specify a target group; rather, they broadly indicated being implemented in Indigenous or Aboriginal communities. Fifteen interventions (44%) were implemented in provinces in Eastern Canada (Newfoundland and Labrador, New Brunswick, Nova Scotia, Ontario, Prince Edward Island, Quebec), 13 interventions (38%) in provinces in Western Canada (Alberta, British Columbia, Manitoba, and Saskatchewan), and four (12%) in the Territories (Northwest Territories, Yukon, and Nunavut). One was a national intervention in several provinces (3%), and one was in an unspecified location (3%).

Findings related to the four components of CSH (social and physical environments, teaching and learning, school policy, and partnerships and services) and the four additional key components of Indigenous school-based nutrition interventions that were examined (cultural content, Indigenous control and ownership, funding source, evaluation) are described in detail below.

### Social and physical environment

Thirty-three interventions (97%) included one or more social and physical environment component. Seven interventions (21%) contributed to the social environment by providing healthy eating messages in newsletters or websites, or by displaying posters in the school that promoted healthy eating. For example, the Hillside Elementary School and Greenwood Elementary School Active Schools programs displayed posters in classrooms that promoted healthy lifestyles and sent newsletters home that included healthy recipes [64]. To encourage both healthy relationships and healthy eating, three interventions (9%) included community feasts. Three interventions (9%) offered student cooking classes or community kitchens, where children learned about healthy eating, practiced cooking skills, and enjoyed nutritious meals. In addition, four interventions (12%) included a peer-mentoring component in which younger students learned about healthy eating from older peers. For example, the Aboriginal Youth Mentorship Program (AYMP) was an after-school peer mentoring program that included healthy snack and nutrition education components [40, 44]. Staff modelling was also recommended by one intervention (3%), which specifically encouraged staff to portray and model healthy eating and positive attitudes towards healthy eating.

Most interventions also included physical components that increased students’ access and exposure to healthy food choices. The majority of interventions (*n* = 25, 74%) offered food programs, with some of them offering breakfast, lunch, and snack (*n* = 6, 24%), breakfast solely (*n* = 4, 16%), breakfast and snack (*n* = 1, 4%), breakfast and lunch (*n* = 3, 12%), lunch solely (n = 1, 4%), lunch and snack (n = 1, 4%), and snack solely (*n* = 8, 32%). One intervention (3%) mentioned offering student nutrition programs but did not specify the meal(s) that were included. Furthermore, three interventions (9%) mentioned student access to a canteen stocked with healthy snacks, and two schools (6%) had vending machines with healthy options. Six interventions (18%) included a school or community garden, and five interventions (15%) included a nutrition awareness campaign or contest. For example, Elsipogtog First Nation School in New Brunswick hosted a healthy snack challenge, in which students who ate a fruit or vegetable during snack time were entered into a draw and had a chance to win a fruit basket [43].

### Teaching and learning

One or more teaching and learning components were used in 19 interventions (56%). Fifteen interventions (44%) included a classroom education component in which discussions of healthy food choices were incorporated into the curriculum. The Kahnawake Schools Diabetes Prevention Project (KSDPP) in Quebec, for example, implemented a comprehensive education program for diabetes prevention that included lessons on balanced meals and healthy snacks, the benefits of healthy eating, factors that influence eating habits, and food label reading [65, 71]. Two interventions (6%) incorporated Indigenous land-based learning (i.e., hunting and fishing) into the curriculum. Four interventions (12%) offered a gardening program in which students learned to plant and harvest vegetables and fruits in the community or school gardens. Finally, three interventions (9%) offered professional development opportunities to teachers and staff related to providing nutrition education.

### Policy

Thirteen interventions (*n* = 13, 38%) included a policy component; however, the scope and content of policies was highly variable. Five interventions (15%) banned or actively discouraged junk food items (e.g., high fat and high sugar foods) from being brought to school. For example, ﻿Chief Harold Sappier Memorial Elementary School in New Brunswick discouraged parents from packing foods like potato chips, candy, and pop in student lunches in an effort to eliminate junk food from the school environment [86]. Four interventions (12%) included food policy guidelines that outlined appropriate foods to serve in school food programs or sell in school vending machines. For example, the Kashechewan snack program in Ontario included written guidelines that outlined categories and frequency of foods to be served in the school [47]. Two interventions (6%) stated that they were compliant with national and/or provincial guidelines, and one intervention (3%) mentioned having a healthy food policy but did not provide any details about the policy content. Finally, the nutrition policy implemented as part of KSDPP targeted a wide range of social and environmental factors to promote healthy food choices, including recommendations for staff, classroom celebrations, and eating environments [58].

### Partnerships and services

Twenty interventions (59%) included one or more partnerships and services component(s). Six interventions (18%) included a parent and community engagement component in which school nutrition activities were reinforced and supported by activities that engaged families and the community-at-large. For example, Yukon Food for Learning encouraged volunteer involvement in delivering school nutrition programs [90]. Two interventions (6%) also specifically mentioned engaging with Elders – or persons recognized for their wisdom, experience, and knowledge – who played a role in delivering nutrition education curricula by sharing their knowledge of cultural activities and traditional foods.

Sixteen interventions (47%) included partnerships with local health and social organizations, local businesses, and national health promoting agencies. For example, Zhiiwapenewin Akino’maagewin: Teaching to Prevent Diabetes (ZATPD) in Ontario was implemented in partnership with several schools, local stores, and health and social services in order to extend its reach in the community [52, 53, 76]. Three interventions (9%) also specifically connected with dietitians or nutritionists, who assisted in planning school food program menus or provided individualized counselling for staff, students, and parents.

### Cultural content

Nineteen interventions (56%) included one or more cultural components. Ten interventions (29%) included traditional foods – such as bannock (a quick bread) and wild game meat – in the schools’ food programs or the education curriculum. Four interventions (12%) incorporated traditional Indigenous ways of learning, such as learning through observation and practice, storytelling, and role modeling. Six interventions (18%) mentioned making culturally appropriate adaptations to education curricula and/or having community members review education materials for cultural sensitivity and relevance. Cultural adaptations included using Indigenous characters in stories and incorporating traditional stories and foods in lessons.

### Indigenous control and ownership

Twenty-one interventions (62%) included a component in which the local community was actively involved in developing, implementing, and/or evaluating interventions. Seven interventions (21%) included information regarding programs or services being community initiated, driven, and/or developed. For example, the National Aboriginal Nutrition Program followed a community-led approach in which key stakeholders – including teachers, school staff, parents, and community members – collaboratively coordinated school nutrition activities [36]. Fourteen interventions (41%) specified using participatory models of research (i.e., participatory action research and community-based participatory research) in which academic researchers and community members worked in collaboration. For example, Kipohtakaw Education Centre in Alberta developed, implemented, and evaluated both a school nutrition policy and gardening intervention through a community-based participatory research approach involving an equitable collaboration between community members and University researchers [49, 51, 66, 67, 72, 88, 89].

### Funding source

Twenty interventions (59%) reported one or more sources of funding. Twelve interventions (35%) received funding from donations, sponsorships, or funding from diverse organizations (e.g., corporations, companies, and charitable foundations). Examples included the Heart and Stroke Foundation of Canada, the Danone ﻿Institute of Canada, Canadian Feed the Children, ONEXONE, and Breakfast for Learning. Nine interventions (26%) were supported by research grant funding, including the Canadian Institutes of Health Research and University Departments. Regional and federal funding (e.g., Health Canada’s First Nations and Inuit Health Branch, Yukon Government Department of Education, and the Health and Wellness fund through the Government of the Northwest Territories) supported six interventions (18%). Finally, one intervention (3%) was supported by the operational budget of the local school board.

### Evaluation

Fourteen interventions (41%) performed evaluations to understand the feasibility of interventions, the barriers and enablers of their implementation, and/or their impact and outcome on student knowledge, behavior, and health. For example, the Sandy Lake Health and Diabetes Project (SLHDP) in Ontario completed two evaluations to determine ﻿changes in students’ knowledge, skills, and self-efficacy and behaviors related to diet by collecting anthropometric data and having students complete a questionnaires and dietary recalls [16, 60].

## Discussion

This scoping review provides an overview of school-based nutrition interventions for Indigenous children that have been implemented in Canada as well as a discussion of the components of identified interventions. Most of the interventions found in this review were implemented for First Nations children, as few nutrition interventions were found for Inuit and Métis populations through the search strategy. In addition, most interventions were implemented in Western Canada, and few were found in the Territories. Other reviews have also noted the under-representation of Inuit and Métis within the scientific intervention literature [21, 28], which inadequately reflects the demographic composition of Indigenous peoples in Canada and most likely the range of school nutrition interventions being implemented in Indigenous communities. This finding highlights the need for more evaluation and active knowledge dissemination concerning interventions implemented in Inuit and Métis populations in Canada to build the evidence base concerning the diversity of Indigenous nutrition interventions.

According to the CSH approach, school-based nutrition interventions should include components that promote health and improve access to healthy foods through social and physical environments, teaching and learning, school policy, and partnerships and services [25, 32]. All of these components must be implemented for an intervention to be considered fully comprehensive and to have the most potential to create sustainable changes in the eating habits of children. In addition, school-based nutrition interventions for Indigenous children should include cultural content, community control and ownership, funding, and evaluation to ensure relevancy and sustainability. This review indicates that a minority of school-based nutrition interventions in Indigenous communities are comprehensive, as few included each of the four aspects of CSH and/or the four additional components identified as important in school-based nutrition interventions for Indigenous children.

All but one of the interventions included components related to the social and physical environment, which indicates that this component is important and relevant in Indigenous settings. Interventions targeted the social environment through community feasts, cooking classes, and peer-mentoring, which encouraged both healthy relationships and promoted healthy eating. With respect to the physical environment, the majority of schools offered food programs that supplemented children’s diets with healthy foods. This finding supports previous research demonstrating that most band-operated First Nations schools offer food programs for students [14]. Although most schools provided breakfast, lunch, and/or snacks, the absence of food programs in one-quarter of interventions may reflect the barriers to initiating and sustaining these initiatives in schools for Indigenous children, which include challenges with acquiring adequate infrastructure funding and accessing quality and affordable healthy foods [13, 14]. Comprehensive school-based nutrition interventions for Indigenous children should include a social and physical environment component to provide an environment conducive to healthy eating. However, schools require the resources, facilities, and funding to support such programs.

While the teaching and learning component was incorporated in most interventions, this remains an area for improvement in current and future school-based nutrition interventions for Indigenous children. The incorporation of healthy eating skills and knowledge in the classroom and involvement of teachers in promoting nutrition may serve to reinforce other components of nutrition interventions [94]. Land-based learning (e.g., collecting, preparing and eating traditional food) has also been recognized for its decolonizing role in revitalizing traditional food system knowledge and increasing access to healthy foods [7, 95]. Although some interventions included curriculum that allowed children to experience and understand traditional Indigenous subsistence practices such as hunting, fishing, and gathering, this review indicates that the incorporation of land-based learning in schools was seldom reported. The review also reveals a lack of adequate professional development opportunities and ongoing support related to teaching nutrition. In order to improve this component, teachers need the time and resources to develop and integrate specialized nutrition education into the classroom [94].

The partnerships and services component of CSH was included in approximately two-thirds of interventions. Parent and community engagement is key in the successful implementation of school-based interventions, as it increases the potential for nutrition-related activities at school to be supported at home and for the larger community to support the nutrition needs of children [96]. Thus, interventions benefit from including strategies that establish relationships with parents and the broader community. Elder involvement in interventions is also significant in schools that educate Indigenous children, as they are respected role models for younger generations and their involvement in nutrition education and the promotion of healthy eating can help ensure relevance and long-term sustainability of interventions [97].

Of the four components of CSH, policy was implemented by the fewest number of interventions. Written school nutrition policies are an integral component of CSH interventions, as they establish formal standards for all nutrition-related aspects of the school environment and coordinate other aspects of the CSH intervention (e.g., foods available, lessons included in classroom education, and strategies for community and family involvement) [98]. The most comprehensive and effective policies are written in clear language and consider all aspects of a school nutrition environment to create a standard against which to hold the school community accountable for nutrition-related changes [98, 99]. With the exception of KSDPP, the policies identified and described in this review provided limited guidance and were focused mainly on describing the types of food that were allowed, or not allowed, in the school environment. As such, this is an area requiring particular attention by schools when developing comprehensive nutrition interventions.

In addition to the four recognized components of CSH, this review paid attention to four additional key components (cultural content, Indigenous control and ownership, funding source, and evaluation) that should be considered when developing school-based nutrition interventions for Indigenous children [21, 27, 28, 33]. Given the considerable diversity that exists among Indigenous communities in Canada, interventions need to be tailored to local contexts by including specific cultural content such as traditional foods and Indigenous ways of learning [97]. As just over half of interventions described one or more cultural component, this indicates an area that may require more attention when developing school-based nutrition interventions. However, it is likely that interventions were also inevitably missed in the searches due to the heterogenous nature of Indigenous peoples, the diverse settings that Indigenous children are educated in Canada, and the limited number of interventions described in the scientific literature. As such, the lack of cultural components found in this review may also be related to an under-reporting of local interventions and description of their components in the scientific and grey literature.

Community control and ownership has also been recognized as an essential component of health interventions in Indigenous communities that can assist in ensuring that interventions are relevant to local contexts [28, 33]. Community control and ownership helps to ensure that interventions are adapted to the unique needs of individual communities, and there is evidence that this component results in more effective and sustainable interventions [28, 97]. In this review, nearly two-thirds of interventions mentioned community involvement in the development, implementation, and/or evaluation of school-based nutrition interventions. Participatory research methods were used in many interventions, highlighting the shift from expert driven to community driven and engaged approaches to intervention research that rely heavily on relational and equitable ways of working in partnership [100]. The four interventions that included the four components of CSH and four additional key components used participatory methods of research that involved equitable collaboration between Indigenous community members and university researchers. This indicates that collaborative relationships between community members and researchers may further assist in creating and sustaining comprehensive school-based nutrition interventions by increasing intervention relevance, support, and resources.

Funding is also an important consideration when developing comprehensive school-based nutrition interventions as it takes extensive resources to implement and sustain multiple intervention components. Over half of the interventions mentioned funding from a diverse range of sources. Other research has similarly shown that school interventions in Indigenous communities are funded by a myriad of donors, and lack of funding has been identified as the main barrier to implementing nutrition interventions [14]. Indigenous education systems – especially those in rural and remote areas – experience chronic underfunding and face numerous environmental barriers that may affect their ability to deliver comprehensive school-based nutrition interventions [101]. This review further indicates a gap in the literature related to disclosure of sources of funding and resources to support the development of comprehensive nutrition interventions.

Finally, evaluation has been identified as an important component of interventions to demonstrate effectiveness and support the sustainability of programs and policies. Fewer than half of the interventions in this review reported an evaluation component, which highlights an important gap in the information currently available for school-based nutrition interventions for Indigenous children. The lack of evaluation of interventions limits the transferability of knowledge concerning their key components and marginalizes Indigenous communities from the evidence regarding effective comprehensive school-based nutrition interventions [31, 102]. However, school staff may lack the time, financial resources, or capacity to perform evaluations and transfer knowledge of their interventions beyond the community level. Schools for Indigenous children may thus benefit from collaboration with researchers and other professionals to integrate evaluation into interventions and share knowledge of effective (and ineffective) intervention components in the scientific and/or grey literature.

### Limitations

To minimize the risk of omitting relevant sources of evidence, this review included both scientific and grey literature. Although the inclusion of grey literature expanded our range of interventions, our descriptions of these interventions was nonetheless limited to the information as provided in sources of evidence. As such, it is possible that interventions had additional components that were not identified. A more robust review would necessitate identifying, contacting, and consulting all schools attended by Indigenous children to include all nutrition interventions and their components. A review of this nature would require considerable time and resources and was not possible in this case. Finally, although the review identified interventions that had been implemented, it did not include information on how well the interventions were implemented in practice. For example, an evaluation of KSDPP found that teachers implemented the curriculum and enforced the school nutrition policy to varying degrees [71]. Similarly, the Fort Albany Comprehensive School nutrition program encountered challenges associated with the remoteness of the school, which often necessitated healthy foods to be replaced with less healthy alternatives (e.g., apple juice in place of apples) [13].

## Conclusions

Although many school-based nutrition for Indigenous children provide supportive social and physical environmental elements, the review suggests that interventions can be more comprehensive by incorporating culturally relevant nutrition education and professional development opportunities for teachers, written school nutrition policies to guide nutrition activities and environments, and activities that engage families and community members. Sustainable interventions must be controlled and owned by Indigenous communities and include culturally specific traditional foods and ways of learning. Finally, there is a need to increase intervention evaluation and the sharing of knowledge and resources related to funding. These recommendations may be used by communities, as well as by researchers and professionals working collaboratively with communities, in developing comprehensive school-based nutrition interventions to improve the eating behaviors of Indigenous children.

## Supplementary information

**Additional file 1.** MEDLINE search strategy. Full electronic search strategy for MEDLINE database.

**Additional file 2.** Web search strategy. Full grey literature search strategy.

**Additional file 3.** Extraction grid. Full extraction grid.

**Additional file 4.** Preferred Reporting Items for Systematic reviews and Meta-Analyses extension for Scoping Reviews (PRISMA-ScR) Checklist. PRIMSA ScR Checklist.

## Data Availability

The datasets supporting the conclusions of this article are included within the article (and its additional files).

## Abbreviations

AYMP: Aboriginal Youth Mentorship Program
CSH: Comprehensive School Health
KSDPP: Kahnawake Schools Diabetes Prevention Project
MeSH: Medical Subject Headings
PRISMA-ScR: Preferred Reporting Items for Systematic reviews and Meta-Analyses extension for Scoping Reviews
SLHDP: Sandy Lake Health and Diabetes Project
ZATPD: Zhiiwapenewin Akino'maagewin: Teaching to Prevent Diabetes

## References

[1] Skinner Kelly, Hanning Rhona, Tsuji Leonard (2006). Barriers and supports for healthy eating and physical activity for first nation youths in northern Canada. International Journal of Circumpolar Health.

[2] Willows ND. Determinants of healthy eating in Aboriginal peoples in Canada: the current state of knowledge and research gaps. Can J Public Heal. 2005;96(Suppl 3):S32–6.16042162

[3] Bhawra J, Cooke MJ, Hanning R, Wilk P, Gonneville SLH. Community perspectives on food insecurity and obesity: focus groups with caregivers of Métis and off-reserve First Nations children. Int J Equity Health. 2015;14:96. 10.1186/s12939-015-0232-5 PMC4609156 26475264

[4] Smylie J, Firestone M. The health of Indigenous peoples. In: Raphael D, editor. Social determinants of health: Canadian perspectives. 3rd ed. Toronto: Canadian Scholars’ Press Inc.; 2016. p. 434–66.

[5] Statistics Canada (2017). Aboriginal peoples in Canada: key results from the 2016 Census.

[6] Willows Noreen D., Hanley Anthony J.G., Delormier Treena (2012). A socioecological framework to understand weight-related issues in Aboriginal children in Canada. Applied Physiology, Nutrition, and Metabolism.

[7] Bagelman Caroline (2018). Unsettling Food Security: The Role of Young People in Indigenous Food System Revitalisation. Children & Society.

[8] Downs SM, Arnold A, Marshall D, McCargar L, Raine K, Willows N (2009). Associations among the food environment, diet quality and weight status in Cree children in Quebec. Public Health Nutr.

[9] Gates Allison, Hanning Rhona M., Gates Michelle, Skinner Kelly, Martin Ian D., Tsuji Leonard J. S. (2012). Vegetable and Fruit Intakes of On-Reserve First Nations Schoolchildren Compared to Canadian Averages and Current Recommendations. International Journal of Environmental Research and Public Health.

[10] Gates A., Skinner K., Gates M. (2014). The diets of school-aged Aboriginal youths in Canada: a systematic review of the literature. Journal of Human Nutrition and Dietetics.

[11] Pigford Ashlee-Ann E., Willows Noreen D. (2010). Promoting optimal weights in Aboriginal children in Canada through ecological research. Childhood Obesity Prevention.

[12] Lee RM, Gortmaker SL, Brownson RC, Colditz GA, Proctor EK (2018). Health dissemination and implementation within schools. Dissemination and implementation research in health: translating science to practice.

[13] Gates Allison, Hanning Rhona M., Gates Michelle, McCarthy Daniel, Tsuji Leonard J. S. (2012). Inadequate Nutrient Intakes in Youth of a Remote First Nation Community: Challenges and the Need for Sustainable Changes in Program and Policy. ISRN Public Health.

[14] Assembly of First Nations. An environmental scan of nutrition programs and policies in First Nations schools. Ottawa: Assembly of First Nations; 2008.

[15] Gates Michelle, Hanning Rhona M., Gates Allison, Isogai Andrea, Tsuji Leonard J.S., Metatawabin Joan (2013). A Pilot Comprehensive School Nutrition Program Improves Knowledge and Intentions for Intake of Milk and Milk Alternatives Among Youth in a Remote First Nation. Journal of Nutrition Education and Behavior.

[16] Saksvig BI, Gittelsohn J, Harris SB, Hanley AJG, Valente TW, Zinman B (2005). A pilot school-based healthy eating and physical activity intervention improves diet, food knowledge, and self-efficacy for native Canadian children. J Nutr.

[17] Kulinna Pamela Hodges (2016). School staff perceptions of factors influencing participation in a Whole-of-School initiative in an Indigenous community. Health Education Journal.

[18] Steckler A, Ethelbah B, Martin CJ, Stewart D, Pardilla M, Gittelsohn J (2003). Pathways process evaluation results: a school-based prevention trial to promote healthful diet and physical activity in American Indian third, fourth, and fifth grade students. Prev Med.

[19] Ho L. S. (2006). Development of an integrated diabetes prevention program with First Nations in Canada. Health Promotion International.

[20] Hoelscher DM, Kirk S, Ritchie L, Cunningham-Sabo L, Academy Positions Committee. Position of the academy of nutrition and dietetics: interventions for the prevention and treatment of pediatric overweight and obesity. J Acad Nutr Diet. 2013;113(10):1375–94. 10.1016/j.jand.2013.08.004 24054714

[21] Godin K., Leatherdale S. T., Elton-Marshall T. (2015). A systematic review of the effectiveness of school-based obesity prevention programmes for First Nations, Inuit and Métis youth in Canada. Clinical Obesity.

[22] Naylor PJ, Scott J, Drummond J, Bridgewater L, McKay HA, Panagiotopoulos C. Implementing a whole school physical activity and healthy eating model in rural and remote First Nations schools: a process evaluation of Action Schools! BC. Rural Remote Health. 2010;10(2):1296. 20476839

[23] Fung C, Kuhle S, Lu C, Purcell M, Schwartz M, Storey K (2012). From “best practice” to “next practice”: the effectiveness of school-based health promotion in improving healthy eating and physical activity and preventing childhood obesity. Int J Behav Nutr Phys Act.

[24] Stewart-Brown S (2006). What is the evidence on school health promotion in improving health or preventing disease and, specifically, what is the effectiveness of the health promoting schools approach?.

[25] Veugelers PJ, Schwartz ME (2010). Comprehensive school health in Canada. Can J Public Heal.

[26] Tran BX, Ohinmaa A, Kuhle S, Johnson JA, Veugelers PJ (2014). Life course impact of school-based promotion of healthy eating and active living to prevent childhood obesity. PLoS One.

[27] Towns Claire, Cooke Martin, Rysdale Lee, Wilk Piotr (2014). Healthy Weights Interventions in Aboriginal Children and Youth: A Review of the Literature. Canadian Journal of Dietetic Practice and Research.

[28] Rice Kathleen, Te Hiwi Braden, Zwarenstein Merrick, Lavallee Barry, Barre Douglas Edward, Harris Stewart B. (2016). Best Practices for the Prevention and Management of Diabetes and Obesity-Related Chronic Disease among Indigenous Peoples in Canada: A Review. Canadian Journal of Diabetes.

[29] Arksey H, O’Malley L (2005). Scoping studies: towards a methodological framework. Int J Soc Res Methodol Theory Pract.

[30] Tricco A, Lillie E, Zarin W, O’Brien K, Colquhoun H, Levac D (2018). PRISMA extension for scoping reviews (PRISMA-ScR): checklist and explanation. Ann Intern Med.

[31] Godin K, Stapleton J, Kirkpatrick SI, Hanning RM, Leatherdale ST (2015). Applying systematic review search methods to the grey literature: a case study examining guidelines for school-based breakfast programs in Canada. Syst Rev.

[32] Pan-Canadian Joint Consortium for School Health. What is comprehensive school health?. 2016. http://www.jcsh-cces.ca/index.php/about/comprehensive-school-health. Accessed 1 Dec 2018.

[33] Tagalik S. A framework for Indigenous school health: foundations in cultural principles. Prince George: National Collaborating Centre for Aboriginal Health; 2010.

[34] Peters MDJ, Godfrey CM, Khalil H, McInerney P, Parker D, Soares CB (2015). Guidance for conducting systematic scoping reviews. Int J Evid Based Healthc.

[35] Brait E. Teacher in remote Inuit community teaches students to garden. Star. 2017; https://www.thestar.com/news/canada/2017/04/26/teacher-in-remote-inuit-community-teaches-students-to-garden.html. Accessed 30 Jan 2019.

[36] Canadian Feed the Children (2019). National Aboriginal Nutrition Program - Canada.

[37] Cargo Margaret, Lévesque Lucie, Macaulay Ann C., McComber Alex, Desrosiers Serge, Delormier Treena, Potvin Louise (2003). Community governance of the Kahnawake Schools Diabetes Prevention Project, Kahnawake Territory, Mohawk Nation, Canada. Health Promotion International.

[38] Carpenter Johnson A, Halas J (2011). Rec and read mentor programs. Reclaiming Child Youth.

[39] Carpenter A. Rec and read: stories of an Aboriginal youth mentor program: University of Manitoba. Winnipeg: Master of Arts thesis; 2009.

[40] Carpenter A, Rothney A, Mousseau J, Halas J, Forsyth J. Seeds of encouragement: initiating an Aboriginal youth mentorship program. Can J Nativ Educ. 2008;31(2):51–69.

[41] Chopra G. Royal Eagles support Amiskwaciy lunch program. Windpeaker. 2007:19.

[42] Dyck Fehderau D, Holt NL, Ball GD, Willows ND. Feasibility study of asset mapping with children: identifying how the community environment shapes activity and food choices in Alexander First Nation. Rural Remote Health. 2013;13(2):2289. 23534835

[43] Elsipogtog First Nation School. Elsipogtog First Nation School. 2017. http://www.elsipogtogschool.ca. Accessed 30 Jan 2019.

[44] Eskicioglu P., Halas J., Senechal M., Wood L., McKay E., Villeneuve S., Shen G. X., Dean H., McGavock J. M. (2014). Peer Mentoring for Type 2 Diabetes Prevention in First Nations Children. PEDIATRICS.

[45] Farm to Cafeteria Canada. Learning circles: local healthy foods to school initative expands to four Indigenous communities. 2018. http://www.farmtocafeteriacanada.ca/2017/04/learning-circles-local-healthy-foods-to-school-initiative-expands-to-four-indigenous-communities. Accessed 30 Jan 2019.

[46] Gates A. (2011). A School Nutrition Program Improves Vegetable and Fruit Knowledge, Preferences, and Exposure in First Nation Youth. The Open Nutrition Journal.

[47] Gates M. Investigation of milk and alternatives intake and the impact of school nutrition programs in First Nations schoolchildren: University of Waterloo. Waterloo: Master’s thesis; 2010.

[48] Gates Michelle, Hanning Rhona M., Gates Allison, McCarthy Daniel D., Tsuji Leonard J. S. (2013). Assessing the Impact of Pilot School Snack Programs on Milk and Alternatives Intake in 2 Remote First Nation Communities in Northern Ontario, Canada. Journal of School Health.

[49] Gillies Christina, Farmer Anna, Maximova Katerina, Willows Noreen D. (2018). First Nations students’ perceptions of school nutrition policy implementation: A mixed methods study. Nutrition & Dietetics.

[50] Green M. One in two Indigenous children in danger of going to school hungry. Star. 2018; https://www.thestar.com/vancouver/2018/06/21/one-in-two-indigenous-children-in-danger-of-going-to-school-hungry.html. Accessed 30 Jan 2019.

[51] Hanbazaza Mahitab A., Triador Lucila, Ball Geoff D.C., Farmer Anna, Maximova Katerina, Alexander First Nation, Willows Noreen D. (2015). The Impact of School Gardening on Cree Children's Knowledge and Attitudes toward Vegetables and Fruit. Canadian Journal of Dietetic Practice and Research.

[52] Ho LS. Diabetes prevention in northwestern Ontario First Nations: a multi-institutional program to improve diet and increase physical activity: John Hopkins University. Baltimore: PhD thesis; 2007.

[53] Ho Lara S., Gittelsohn Joel, Rimal Rajiv, Treuth Margarita S., Sharma Sangita, Rosecrans Amanda, Harris Stewart B. (2006). An Integrated Multi-Institutional Diabetes Prevention Program Improves Knowledge and Healthy Food Acquisition in Northwestern Ontario First Nations. Health Education & Behavior.

[54] Inuit Tapiriit Kanatami. School breakfast program – Inuvialuit settlement region. 2018. https://www.itk.ca/itk_initiative_type/school-food-programs. Accessed 30 Jan 2019.

[55] Inuit Tapiriit Kanatami. Nunavik breakfasts. 2018. https://www.feedingnunavut.com/inuit-community-based-food-initiatives. Accessed 30 Jan 2019.

[56] Isogai AD, Gates A, Gates M, Hanning RM, Tsuji LJS. A qualitative evaluation of the efficacy of the delivery of the educational component of a nutrition program in a remote First Nation community. Pimatisiwin A J Aborig Indig Community Heal. 2012;9(2):349–62.

[57] Jimenez M.Michelle, Receveur Olivier, Trifonopoulos Mary, Kuhnlein Harriet, Paradis Gilles, Macaulay Ann C. (2003). Comparison of the dietary intakes of two different groups of children (grades 4 to 6) before and after the Kahnawake Schools Diabetes Prevention Project. Journal of the American Dietetic Association.

[58] Kahnawake Elementary School. The wellness policy for Kahnawake Elementary Schools: nutrition. http://www.neola.com/miamidade-fl/search/policies/po8510.htm. Accessed 30 Jan 2019.

[59] Kahnawake Schools Diabetes Prevention Project. Kahnawake Schools Diabetes Prevention Project (KSDPP) website. http://www.ksdpp.org. Accessed 1 Mar 2019.

[60] Kakekagumick KE, Goodman S, Gittelsohn J, Manokeesic G, Saksvig B, Harris SB, et al. Sandy Lake Health and Diabetes Project: a community-based intervention targeting type 2 diabetes and its risk factors in a First Nations community. Front Endocrinol. 2013;4:1–9. 10.3389/fendo.2013.00170 PMC3824247 24302919

[61] Khayyat Kholghi Maedeh, Bartlett Gillian, Phillips Morgan, Salsberg Jon, McComber Alex M, Macaulay Ann C (2017). Evaluating an Indigenous health curriculum for diabetes prevention: engaging the community through talking circles and knowledge translation of results. Family Practice.

[62] Lickers A. Longhouse and greenhouse: searching for food security in a community based research project: Royal Roads University. Victoria: Master’s thesis; 2015.

[63] Macaulay Ann C., Ing Amy., Salsberg Jon., McGregor Amelia., Saad-Haddad Chantal., Rice Joyce., Montour Lois., Gray-Donald Katherine. (2007). Community-Based Participatory Research: Lessons From Sharing Results With the Community: Kahnawake Schools Diabetes Prevention Project. Progress in Community Health Partnerships: Research, Education, and Action.

[64] Matsumura L. The journey toward comprehensive school health within an Aboriginal community: Brock University. St. Catharines: Master’s thesis; 2009.

[65] McComber AM. Improving nutrition through community partnerships at Indigenous schools: the experience of the Kahnawake Schools Diabetes Prevention Project (KSDPP). 2015.https://changingthemenu.org/sites/default/files/upload/ksdpp_change_the_menu_presentation_mccomber_151113.pdf. Accessed 30 Jan 2019.

[66] Murray K. School staff identified barriers, facilitators, and perceptions of implementing a school nutrition policy in a First Nation community school: University of Alberta. Edmonton: Master’s thesis; 2016.

[67] Murray Kris, Research Committee Alexander, Farmer Anna, Maximova Katerina, Willows Noreen (2017). "It's huge in First Nation culture for us, as a school, to be a role model": Facilitators and Barriers Affecting School Nutrition Policy Implementation in Alexander First Nation. International Journal of Indigenous Health.

[68] Noojmowin Teg. Child nutrition program. http://www.noojmowin-teg.ca/SitePages/Child Nutrition Program.aspx. Accessed 30 Jan 2019.

[69] Oosman SN. Kica-Wasimisinanahk Miyo-ayawin - our children’s health: promoting physical activity and nutrition through a health promoting school-based intervention in a Métis community: University of Saskatchewan. Saskatoon: PhD thesis; 2012.

[70] Pan-Canadian Joint Consortium for School Health. School health promotion in Nunavut. 2009. http://jcsh-cces.ca/upload/School Health Promotion in Nunavut.pdf. Accessed 30 Jan 2019.

[71] Paradis G. (2005). Impact of a Diabetes Prevention Program on Body Size, Physical Activity, and Diet Among Kanien'keha:ka (Mohawk) Children 6 to 11 Years Old: 8-Year Results From the Kahnawake Schools Diabetes Prevention Project. PEDIATRICS.

[72] Pigford AAE, Ball GDC, Plotnikoff RC, Arcand E, Alexander First Nation, Dyck Fehderau D, et al. Community-based participatory research to address childhood obesity: experiences from Alexander First Nation in Canada. Pimatisiwin A J Aborig Indig Community Heal. 2013;11(2):171–86.

[73] Priest A (2006). Nunavut’s drop the pop campaign. Can Nurse.

[74] Receveur Olivier, Morou Karimou, Gray-Donald Katherine, Macaulay Ann C. (2008). Consumption of Key Food Items Is Associated with Excess Weight among Elementary-School–Aged Children in a Canadian First Nations Community. Journal of the American Dietetic Association.

[75] Ronsley Rebecca, Lee Andrew S., Kuzeljevic Boris, Panagiotopoulos Constadina (2013). Healthy Buddies™ Reduces Body Mass Index Z-Score and Waist Circumference in Aboriginal Children Living in Remote Coastal Communities. Journal of School Health.

[76] Rosecrans A. M., Gittelsohn J., Ho L. S., Harris S. B., Naqshbandi M., Sharma S. (2007). Process evaluation of a multi-institutional community-based program for diabetes prevention among First Nations. Health Education Research.

[77] Saksvig BI. Diabetes prevention among First Nations school children in Sandy Lake, Ontario: evaluation of a culturally appropriate school-based nutrition and physical activity intervention: John Hopkins University. Baltimore: PhD thesis; 2002.

[78] Salmon L. Contribution of foods to nutrient intakes of grades 4–6 students participating in Kahnawake Schools Diabetes Prevention Project 1994, 1998 and 2002: McGill University. Montreal: Master’s thesis; 2004.

[79] Sexsmith P (2007). New school immerses students in their culture. Windspeaker..

[80] Skinner K, Hanning RM, Metatawabin J, Martin ID, Tsuji LJS. Impact of a school snack program on the dietary intake of grade six to ten First Nation students living in a remote community in northern Ontario, Canada. Rural Remote Health. 2012;12(3):1–17. 22909226

[81] Skinner Kelly, Hanning Rhona M., Sutherland Celine, Edwards-Wheesk Ruby, Tsuji Leonard J. S. (2012). Using a SWOT Analysis to Inform Healthy Eating and Physical Activity Strategies for a Remote First Nations Community in Canada. American Journal of Health Promotion.

[82] St-Onge J. Indigenous high school students feed schoolchildren in Maskwacis. CBC News 2018. https://www.cbc.ca/news/canada/edmonton/universal-food-program-maskwacis-school-student-ermineskin-1.4880982. Accessed 30 Jan 2019.

[83] Teach for Canada. Eabametoong First Nation. 2017. https://teachforcanada.ca/en/wp-content/uploads/2017/09/Eabametoong-WEB.pdf. Accessed 30 Jan 2019.

[84] Thompson Heather A., Mason Courtney W., Robidoux Michael A. (2018). Hoop House Gardening in the Wapekeka First Nation as an Extension of Land-Based Food Practices. ARCTIC.

[85] Foodshare. FoodShare Toronto: Aboriginal school program. http://ademe.innovationsociale.org/en/project/foodshare-toronto-aboriginal-school-program. Accessed 30 Jan 2019.

[86] Tombs D (2004). School gives a BOOST to student well-being. Wind Publ.

[87] Tomlin Dona, Naylor PJ, McKay Heather, Zorzi Alexandra, Mitchell Marc, Panagiotopoulos Constadina (2012). The impact of Action Schools! BC on the health of Aboriginal children and youth living in rural and remote communities in British Columbia. International Journal of Circumpolar Health.

[88] Triador L. The effect of school gardening and a healthy snack program on First Nations children’s knowledge and attitudes about vegetables and fruit, and their consumption of these foods at home: University of Alberta. Edmonton: Master’s thesis; 2013.

[89] Triador Lucila, Farmer Anna, Maximova Katerina, Willows Noreen, Kootenay Jody (2015). A School Gardening and Healthy Snack Program Increased Aboriginal First Nations Children's Preferences Toward Vegetables and Fruit. Journal of Nutrition Education and Behavior.

[90] Yukon Food for Learning. Schools & communities. 2016. http://www.yukonfoodforlearning.ca. Accessed 30 Jan 2019.

[91] Acadia First Nation (2016). Acadia First Nation youth center after school program.

[92] Adams A, Receveur O, Mundt M, Paradis G, Macaulay AC. Healthy lifestyle indicators in children (grades 4 to 6) from the Kahnawake Schools Diabetes Prevention Project. Can J Diabetes. 2005;29(4):403–9.

[93] Athabasca Basin Development, ONEXONE. ONEXONE First Nations school breakfast program launches in four Sask. schools. 2016. http://athabascabasin.ca/wp-content/uploads/2016/04/Media-Release-ONEXONE-ABD-April-14.pdf. Accessed 30 Jan 2019.

[94] Cargo M, Salsberg J, Delormier T, Desrosiers S, Macaulay AC (2006). Understanding the social context of school health promotion program implementation. Health Educ.

[95] Wesche Sonia D., O'Hare-Gordon Meagan Ann F., Robidoux Michael A., Mason Courtney W. (2016). Land-Based programs in the Northwest Territories: Building Indigenous food security and well-being from the ground up. Canadian Food Studies / La Revue canadienne des études sur l'alimentation.

[96] Gillies C, Alexander Reseach Committee, Farmer A, Maximova K, Willows ND. Alexander First Nations parents’ perceptions of a school nutrition policy. Can J Diet Pract Res. 2019;12:1–6. 10.3148/cjdpr-2019-026 31512509

[97] Oosman S, Smylie J, Humbert L, Henry C (2016). Métis community perspectives inform a school-based health promotion intervention using participatory action. Engag Sch J.

[98] McKenna ML (2010). Policy options to support healthy eating in schools. Can J Public Heal..

[99] Schwartz M, Henderson K, Falbe J, Novak S, Wharton C, Long M (2012). Strength and comprehensiveness of district school wellness policies predict policy implementation at the school level. J Sch Health.

[100] Gokiert RJ, Willows ND, Georgis R, Stringer H, Alexander Research Committee. Wâhkôhtowin: the governance of good community–academic research relationships to improve the health and well-being of children in Alexander First Nation. Int Indig Policy J. 2017;8(2):1–20.

[101] National Collaborating Centre for Aboriginal Health. Education as a social determinant of First Nations, Inuit and Métis health. Prince George: National Collaborating Centre for Aboriginal Health; 2017.

[102] Saini M, Quinn A. A systematic review of randomized controlled trials of health related issues within an Aboriginal context. Prince George; 2013. http://www.ccnsa-nccah.ca/Publications/Lists/Publications/Attachments/94/RCT_EN_web.pdf. Accessed 15 Feb 2019.

