# Supplementary material for: School-based nutrition interventions for Indigenous children in Canada: a scoping review
Source: BMC Public Health. 2020 Jan 6;20:11. doi: 10.1186/s12889-019-8120-3 (PMC6945607; doi:10.1186/s12889-019-8120-3)
Supplement: Supplementary file 1 — Additional file 1. MEDLINE search strategy. Full electronic search strategy for MEDLINE database. [file 12889_2019_8120_MOESM1_ESM.docx]

Additional File 1. MEDLINE search strategy

| Number | Search | Results |
| --- | --- | --- |
| 1 | exp Indians, North American/ | 13760 |
| 2 | (aboriginal* or first nation* or Premiere Nation or native* or Inuit* or Metis or Indigenous or FNIM or Amerindian or Indigenous or autochtone* or Mixed-blood* or Half Breed* or halfbreed* or Cree or Blackfoot or Blackfeet or Ojibwe or Ojibwa or Oji-Cree or Saulteaux or Anishinaabe or Assiniboin* or Anishinaabeg* or Mischif or Mitchif or Metif or Metchif or Dene or Wakashan or Athapaskan or Athapascan or Eskimo* or Esquimau* or Innu or Inuk or Innus or Montagnais or Maliseet or Naskapi or Mikmaq or Micmac or Mic mac or Migmaw or Micmaw or Beothuk* or "Gwich'in" or Inuvialuktun or Algonquin* or Algonquian* or Chipewyan or Tlingit or Mohawk or ((Native or Indian or Indians) adj1 (man or men or women or woman or boy* or girl* or adolescent* or youth or youths or person* or adult or people* or Indian or Indians or Nation* or tribe* or tribal or band or bands or population))).mp. | 311225 |
| 3 | 1 or 2 | 311225 |
| 4 | exp Schools/ | 109191 |
| 5 | (kindergarden* or kindergarten* or elementary or primary school* or nursery school* or preschool* or pre-school* or pre school or pre-primary school or pre primary school or playschool* or secondary school* or high school* or senior high school* or "k-12" or junior high school* or middle school* or junior high school or residential school* or school*).mp. | 1162659 |
| 6 | 4 or 5 | 1193968 |
| 7 | exp Food/ or exp Eating/ or exp Diet/ or exp Food Habits/ or food services/ or menu planning/ or nutritional status/ or Energy Intake/ | 1504860 |
| 8 | (nutrition* or food* or eat or eats or eating or diet or diets or dieting or nutrient* or feed or feeding or menu or menus or cafeteria* or confection?ry or canteen* or vegetable* or fruit or fruits or breakfast or lunch or meal or meals or garden* or snack or snacks).ti,ab. | 1217544 |
| 9 | 7 or 8 | 2213559 |
| 10 | canada/ or alberta/ or british columbia/ or manitoba/ or new brunswick/ or "newfoundland and labrador"/ or northwest territories/ or nova scotia/ or nunavut/ or ontario/ or prince edward island/ or quebec/ or saskatchewan/ or yukon territory/ | 148109 |
| 11 | (canad* or canadian* or alberta* or british columbia* or saskatchewan* or manitoba* or ontario or ontarian* or quebec or quebecois or new brunswick* or nova scotia* or prince edward island or newfoundland* or labrador* or nunavut or nwt or northwest territories or yukon*).mp,in. | 837346 |
| 12 | 10 or 11 | 837346 |
| 13 | 3 and 6 and 9 and 12 | 304 |
