# Supplementary material for: School-based nutrition interventions for Indigenous children in Canada: a scoping review
Source: BMC Public Health. 2020 Jan 6;20:11. doi: 10.1186/s12889-019-8120-3 (PMC6945607; doi:10.1186/s12889-019-8120-3)
Supplement: Supplementary file 2 — Additional file 2. Web search strategy. Full grey literature search strategy. [file 12889_2019_8120_MOESM2_ESM.docx]

Additional File 2. Web Search Strategy

| # | **Search query** | **# of results retrieved** | **# of results screened** | **# of new potentially relevant records** |
| --- | --- | --- | --- | --- |
| 1 | "School" AND "Indigenous" AND ("nutrition" OR "food") | ~79,000,000 | 100 | 7 |
| 2 | “School” AND “First nation” AND (“nutrition” OR “food”) | ~2,590,000 | 100 | 12 |
| 3 | “School” AND “Aboriginal” AND (“nutrition” OR “food”) | ~30,600,000 | 100 | 3 |
| 4 | “School” AND “Native” AND (“nutrition” OR “food”) | ~275,000,000 | 100 | 0 |
| 5 | “School” AND “Indian” AND (“nutrition” OR “food”) | ~388,000,000 | 100 | 0 |
| 6 | “School” AND “Metis” AND (“nutrition” OR “food”) | ~1,200,000 | 100 | 2 |
| 7 | “School” AND “Inuit” AND (“nutrition” OR “food”) | ~3,000,000 | 100 | 5 |
|  |  |  | Total | 29 |
